# Persistence of Norovirus GII Genome in Drinking Water and Wastewater at Different Temperatures

**DOI:** 10.3390/pathogens6040048

**Published:** 2017-10-11

**Authors:** Ari Kauppinen, Ilkka T. Miettinen

**Affiliations:** Department of Health Security, Expert Microbiology Unit, National Institute for Health and Welfare, P.O. Box 95, FI-70701 Kuopio, Finland; ilkka.miettinen@thl.fi

**Keywords:** drinking water, wastewater, microbial contamination, norovirus, persistence

## Abstract

Human norovirus (NoV) causes waterborne outbreaks worldwide suggesting their ability to persist and survive for extended periods in the environment. The objective of this study was to determine the persistence of the NoV GII genome in drinking water and wastewater at three different temperatures (3 °C, 21 °C, and 36 °C). The persistence of two NoV GII inoculums (extracted from stool) and an indigenous NoV GII were studied. The samples were collected for up to one year from drinking water and for up to 140 days from wastewater. Molecular methods (RT-qPCR) were used to assess the decay of the NoV genome. Decay rate coefficients were determined from the fitted decay curves using log-linear and/or non-linear model equations. Results showed significant differences in the decay kinetics of NoV genome between the temperatures, matrices, and virus strains. The persistence of NoV was higher in drinking water compared to wastewater, and the cold temperature assisted persistence at both matrices. Differences between the persistence of NoV strains were also evident and, particularly, indigenous NoVs persisted better than spiked NoVs in wastewater. The decay constants obtained in this study can be utilized to assess the fate of the NoV genome in different water environments.

## 1. Introduction

Human norovirus (NoV) is one of the most common waterborne pathogens causing acute gastroenteritis worldwide [1,2]. NoVs end up in the environment mainly through wastewater discharge. The environmental transmission of NoVs via water may occur directly using contaminated drinking water [3], or indirectly through recreational activities [4,5], consumption of food produced with contaminated irrigation water [6], or from contaminated shellfish harvesting areas [7]. The occurrence of NoV outbreaks related to water demonstrates that these viruses present in the environment outside the host can survive and stay infectious for a substantial time.

Each microbe has its own characteristic survival and behavioral properties that are highly responsible for the microbe’s capability to cause water-related problems. Overall, waterborne viruses (such as NoV, rotavirus, and adenovirus) are considered to show high persistence in water [8]. The virus decay in water is a complex process expected to be influenced by site-specific environmental conditions, such as the temperature, exposure to sunlight, organic matter content, the presence of indigenous microorganisms, and the physical and chemical water properties [9,10,11]. The understanding of the decay rates of viruses in water has an important role in water safety assessments. The management of contamination cases, as well as specific modelling and risk assessment scenarios, e.g., related to the transport and fate of viruses in the water environment, requires information regarding their survival.

Current detection methods for NoV rely on genome detection since, despite recent progress [12,13], they cannot be grown in simple culture systems. The limitation of genome-based molecular methods is the detection of both infectious and noninfectious virus particles. Despite the many efforts, none of the potential molecular-based infectivity assays have been universally accepted as effective [14,15]. Therefore, the health authorities and decision-makers still need to base their decisions largely on the presence of the genome without the knowledge about the infectivity of NoV.

Previous studies have shown the high persistence of NoV in the water environment [16,17,18,19]. However, information regarding the dependency of NoV genome persistence on different temperatures, water matrices, and NoV strains is still limited. The objective of this study was to examine NoV genome persistence in drinking water and wastewater at 3 °C, 21 °C, and 36 °C, in dark conditions.

## 2. Results

### 2.1. Persistence of the NoV Genome in Drinking Water at Different Temperatures

**Figure 1:**
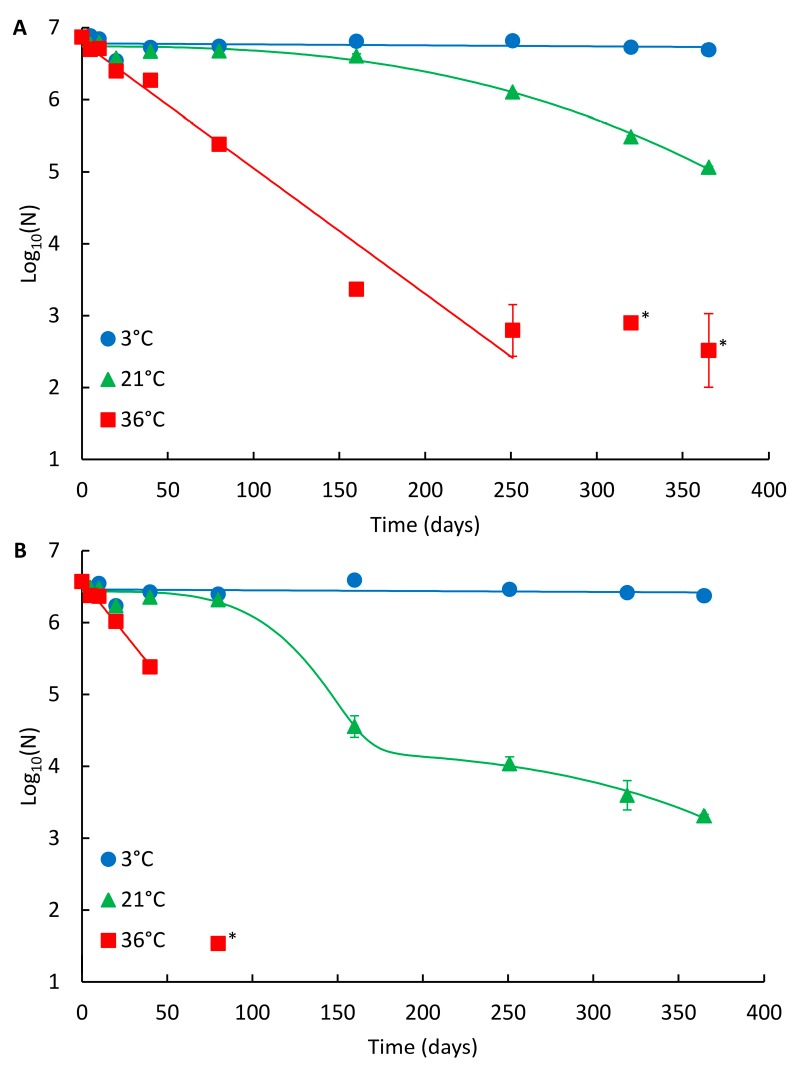
Persistence of the NoV genome in drinking water at different temperatures. (**A**) NoV GII_A and (**B**) NoV GII_B. Identified curves represent the modelled decay of best fit. At 3 °C and 36 °C, log-linear modelled curves are presented, and at 21 °C Weibull and double Weibull modelled curves for GII_A and GII_B are shown, respectively. Error bars show the standard deviation for duplicate extractions. Below limit of quantitation (LOQ) results are shown with an asterisk (*), but not fitted in curves.

The decay curves of NoV GII_A and GII_B in drinking water are presented in Figure 1A,B, respectively, and the summary of the modelling results in Table 1. For the spiked NoV strains (both assigned as recombinant GII.Pg/GII.1), the persistence was highest at 3 °C and lowest at 36 °C (Figure 1; Table 2). At 3 °C, no reduction was observed during one year study and no statistical difference in persistence between the two NoV strains was noted. At 21 °C, both strains were clearly detectable throughout the whole one-year study period (Figure 1). The persistence of GII_A and GII_B was comparable during the first 80 days at 21 °C (log_10_ reduction 0.2 and 0.3, respectively, *p* = 0.059). Subsequently, GII_A persisted better showing non-linear Weibull decay model compared to GII_B showing the double Weibull decay model achieving 1.8 and 3.3 log_10_ reductions, respectively (*p* = 0.001). At 36 °C, log-linear decay was observed for both strains and the persistence of GII_A and GII_B was comparable during the first 20 days (log_10_ reduction 0.5 and 0.6, respectively, *p* = 0.028). After 20 days, GII_A clearly persisted better and was detectable the whole study period compared to GII_B (*p* < 0.001), which was not detected after 160 days at 36 °C.

### 2.2. Persistence of the NoV Genome in Wastewater at Different Temperatures

**Figure 2:**
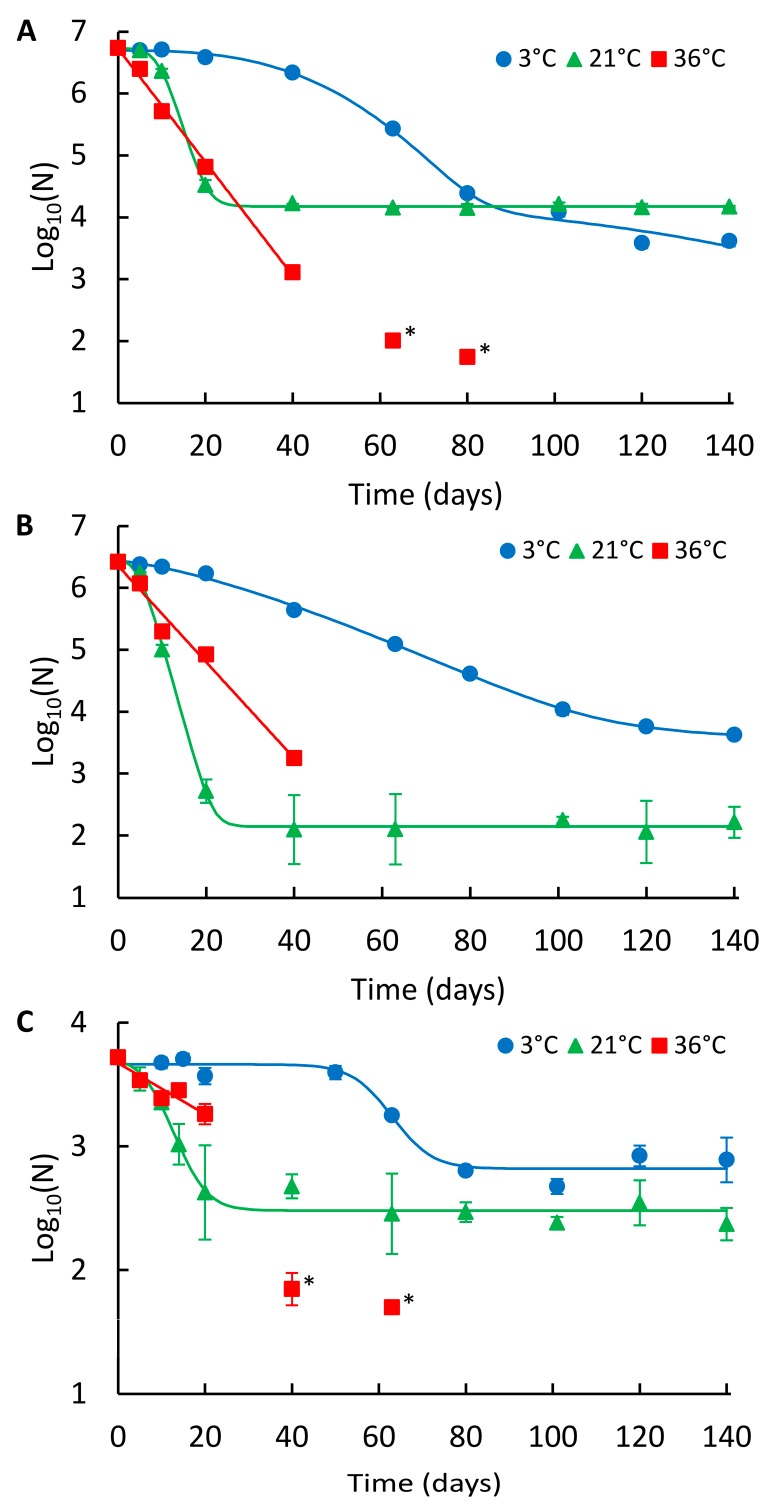
Persistence of the NoV genome in wastewater at different temperatures. (**A**) NoV GII_A, (**B**) NoV GII_B, and (**C**) NoV GII_ind. Identified curves represent the modelled decay of best fit. At 3 °C double Weibull modelled curves for GII_A and GII_B, and log-linear shoulder tail modelled curve for GII_ind are shown. At 21 °C, log-linear shoulder tail and at 36 °C, log-linear modelled curves are presented. Error bars show the standard deviation for duplicate extractions. Below LOQ results are shown with an asterisk (*), but not fitted in curves.

The decays of NoV GII_A, GII_B, and indigenous GII (GII_ind) in wastewater are presented in Figure 2A–C, respectively, and the summary of the modelling results is shown in Table 1. In wastewater, the persistence of NoV was also highest at 3 °C and lowest at 36 °C, except for GII_A, which showed the highest long-term persistence at 21 °C (Figure 2A; Table 2). At 3 °C, GII_A and GII_B showed a non-linear double Weibull decay model and GII_ind log-linear shoulder tail decay model (Table 1). GII_ind was more persistent (log_10_ reduction 0.8) compared to GII_A and GII_B, whose reductions did not differ statistically from each other during the 140-day study at 3 °C (log_10_ reductions 3.1 and 2.8, respectively, *p* = 0.27). At 21 °C, the log-linear shoulder tail decay model was applied for all NoV strains. The persistence of all three strains differed statistically from each other; GII_ind was the most persistent, followed by GII_A and GII_B (log_10_ reductions 1.3, 2.6 and 4.2, respectively). At 36 °C, log-linear decay was observed for all strains and the numbers were decayed below LOD. GII_ind showed higher persistence compared to GII_A and GII_B, whose persistence was not statistically different from each other during the first 40 days (*p* = 0.18).

### 2.3. Effect of the Matrix on NoV Genome Persistence

A comparison of the decay rates showed that both spiked inoculums, GII_A and GII_B, persisted better in drinking water compared to wastewater at each temperature (Table 2).

## 3. Discussion

The results clearly show the effect of temperature, water matrix, and NoV strain on genome persistence. Tests were carried out at three temperatures in order to simulate conditions commonly found in water environments (3 °C and 21 °C) (groundwater, surface water, wastewater, and drinking water), as well as to demonstrate the effect of high temperature (36 °C) representing extreme environmental conditions. NoV persisted longer at cold temperatures in both drinking and wastewater. This observation is consistent with the previous studies showing the temperature dependency of NoV genome persistence. Bae and Schwab [16] and Ngazoa et al. [20] showed that the NoV genome persisted better in different waters at 4 °C than at 25 °C. Similarly, Skraber et al. [18] showed that indigenous NoV GI persisted better in wastewater at 4 °C than at 20 °C. Moreover, Liu et al. [21] showed that the order of persistence of NoV genome in phosphate-buffered saline (PBS) was: 4 °C > RT > 37 °C. 

The decay rates of NoVs used in this study were comparable or lower than those presented in previous studies (Table S1). However, the comparison of the decay rates between different studies is challenging due to differences in experimental conditions, such as the test water properties and the status of the studied NoV strain, which may affect the persistence of a virus genome. In spike tests, the pre-study storage conditions, length of preservation, and the preparation method of the inoculum, presumably have an effect on the results.

In addition to temperature, the matrix was found to have a significant role in the persistence of the NoV genome. Virus decay appeared to occur faster in wastewater than in drinking water. In drinking water, no reduction in genome numbers was observed at 3 °C and NoV was also detectable at 21 °C and 36 °C throughout the whole one-year follow-up period. In contrast to drinking water, significantly higher log_10_ reductions were noted in wastewater during the 140-day study. This is probably due to the higher presence of organic matter and indigenous microorganisms in wastewater, which may have negative effects on NoV persistence [8,9,10,11]. This finding of better persistence of NoV in clean water is consistent with the previous studies examining the persistence of the NoV genome in different water matrices [16,20]. Overall, viruses have been shown to be more persistent in simple (e.g., drinking water) than in complex matrices (e.g., wastewater) [22].

In this study, the persistence of two NoV strains (both GII.Pg/GII.1) were examined in drinking water (GII_A, GII_B) and three in wastewater (GII_A, GII_B, and GII_ind). GII_A and GII_B persisted similarly in both drinking water and wastewater at 3 °C, as well as in wastewater at 36 °C. However, GII_A was found to be more persistent than GII_B in drinking water (after 80 days) and wastewater at 21 °C, as well as in drinking water (after 20 days) at 36 °C. The observed differences between the decay of GII_A and GII_B inoculums at 21 °C and 36 °C may be due to exposure of GII_B to pre-study environmental stress. At that time, putatively, three freeze-thaw cycles occurred, which may have influenced GII_B stability. However, previous study has shown that NoV is resistant to freezing and thawing [23]. Other explanations include the different status of viruses at the time of stool sample collection, as well as the other contents of the stool, such as antiviral compounds and enzymes (nucleases and proteases) present in inoculums, which may have had an effect on the decay.

In wastewater, indigenous GII (GII_ind) persisted better compared to spiked inoculums (GII_A and GII_B) at each temperature. This may be due to four to five years’ storage of spiked viruses prior to study. Moreover, age of indigenous NoVs was unknown and it is possible that only the more persistent population was present at the start of the study. The GII genotype of indigenous NoV may have also been more resistant compared to the GII.Pg/GII.1 genotype of spiked inoculums. Unfortunately, the typing of the wastewater strain was unsuccessful.

To date, many of the persistence studies with NoV have been performed with the GI.1 genotype, [16,19,21], initially prepared in the challenge study in 1997 from over 25-year-old primary inoculum [24]. The comparable or higher persistence of NoVs observed in this study compared to GI.1 may be due to differences in experimental conditions discussed above. In addition, the evolution of NoV during the past 25 years may have improved the environmental persistence of virus, especially considering the large epidemics occurred during 2000. The recombinant GII.Pg/GII.1 NoV, used in this study, have been recently described in multiple outbreaks [25,26,27,28].

The simple first-order log-linear regression model is commonly used to describe NoV genome persistence [16,18,21]. In this study, a log-linear model was observed to fit best for the decay of NoV only at 36 °C. However, at lower temperatures (3 °C and 21 °C), non-linear models produced better fits for the decay curves. At these lower temperatures, the log-linear shoulder tail, Weibull, and double Weibull models were applied to obtain the best fit for the experimental data. This agrees with previous studies where non-linear modelling has been used successfully to describe the thermal persistence of viruses [17,29,30,31]. As shown in our study, sufficient follow-up time is required to detect the non-linear decay. Noteworthy, the decay of GII_A and GII_B in wastewater at 3 °C could also have showed a tailing effect if continued longer than 140 days. Experimental periods from three up to seven weeks [16,18,21] may be insufficient to describe the long-term persistence of the NoV genome.

The long persistence of the NoV genome, especially at lower temperatures, may cause prolonged outbreak management cases as shown in a recent study [32]. In the risk assessments related to outbreak control and management, molecular methods (RT-qPCR) are commonly used for NoV detection without the knowledge of the infectious state of the virus. In drinking water, it may be reasonable to judge the water containing even traces of the NoV genome as non-suitable for human consumption. However, results based on the molecular detection of NoV from environmental samples should be interpreted carefully and in the context of available epidemiological or clinical information [33]. The results of this study emphasize the need and importance of practical and reliable infectivity assay for NoV to reveal the actual infectious risk related to long-term genome persistence. 

## 4. Materials and Methods 

### 4.1. Viruses and Water Matrices

Two NoV GII (GII.Pg/GII.1) inoculums used in this study are described in Table 3. Inoculums were extracted from the human stools stored at −20 °C by making 10–20% (*w*/*v*) suspension in nuclease free water. The suspension was centrifuged at 10,000× *g* for 2 min and the supernatant was used immediately or stored at −75 °C. 

Tests were carried out in drinking water and wastewater matrices. The general physical-chemical properties of the drinking water and wastewater used in this study are presented in Table 4. Drinking water was the tap water of the city of Kuopio, Finland, where the chlorine was quenched with sodium thiosulfate prior to the experiments. Wastewater was settled influent taken from the distribution box after a three-tank septic system of a private onsite wastewater treatment system serving five people (two adults and three children).

### 4.2. Experimental Design and Sampling

Tests were carried out in 15 mL polypropylene tubes coated with aluminum foil to obtain dark conditions. Tubes were incubated at three different temperatures; in a refrigerated room at 3 °C, in a laboratory at 21 °C (RT) and in a heat-controlled room at 36 °C. Temperature was monitored every 5 min with the automated monitoring system (Labo Line). In drinking water, the average temperatures and standard deviations were 3.2 ± 0.8, 21.0 ± 0.8, and 35.9 ± 0.1, and in wastewater was 3.0 ± 0.8, 20.9 ± 0.3, and 35.8 ± 0.1.

In drinking water experiment, 600 µL of NoV inoculum was spiked in 30 mL of Kuopio tap water, separately for GII_A and GII_B, and the mixtures were divided into three temperature tests. The initial numbers in drinking water experiment for GII_A and GII_B were 7.4 × 10^6^ genome copies (GC) mL^−1^ and 3.7 × 10^6^ GC mL^−1^, respectively. In wastewater experiment, similarly, 300 µL of the inoculum was spiked in 30 mL of wastewater, separately for GII_A and GII_B, and the mixtures, as well as non-spiked wastewater sample for the indigenous GII (GII_ind) test, were divided into three temperature tests. The initial numbers of GII_A, GII_B, and GII_ind in wastewater experiment were 5.4 × 10^6^ GC mL^−1^, 2.6 × 10^6^ GC mL^−1^, and 5.2 × 10^3^ GC mL^−1^, respectively. In wastewater test calculations, the numbers of GII_ind were subtracted from the numbers of spiked GII_A and GII_B.

Duplicate samples were taken after 0, 5, 10, 20, 40, 80, 160, 251, 320, and 365 days in drinking water experiment and after 0, 5, 10, 14 (15), 20, 40 (50), 63, 80, 101, 120, and 140 days in wastewater experiment from the beginning of the experimental work. Sample tubes were vortexed briefly prior to sampling and opened only in sterile conditions under a laminar flow hood to prevent microbial contamination. The samples were immediately subjected to RNA extraction.

### 4.3. Quantitative Detection of NoV

Viral RNA was extracted from a 200 µL sample using the High Pure Viral RNA Kit according to the manufacturer’s instructions (Roche Diagnostics GmbH, Mannheim, Germany) and stored at −75 °C. NoV GII was detected using the forward primer QNIF2d [36], reverse primer (COG2R), and probe (RING2-TP) [37], except for the black hole quencher (BHQ) used at the 3′ end of the probe. Amplification reaction mixtures contained 6.25 µL 4X TaqMan^®^ Fast Virus 1-Step Master Mix (Thermo Fisher Scientific, Austin, TX, USA), 0.4 mmol L^−1^ primers, 0.2 mmol L^−1^ probe, and 5 µL of RNA sample or control in a final volume of 25 µL. The real-time RT-qPCR assays were carried out using QuantStudio 6 Flex Real-Time PCR System (Applied Biosystems, Foster City, CA, USA) by running at 50 °C for 5 min and 95 °C for 20 s, followed by 45 cycles at 95 °C for 15 s and 60 °C for 1 min. All samples were run with undiluted and 10-fold dilutions without technical replicates. Quantitation was made by comparing the Ct values of the sample to the serially-diluted standard curve included in each run. Standard curves were generated using gBlocks^®^ Gene Fragments (Integrated DNA Technologies, Leuven, Belgium) containing the sequences for the target amplicon. Negative extraction control (nuclease free water) was included in every batch of extractions. GII_A stored in drinking water at 4 °C was used as positive extraction control in drinking water experiment, and in wastewater experiment with every batch of new reagents. Inhibition in RT-qPCR was assessed by using the 10-fold dilution results in calculations if inhibition was detected.

### 4.4. Sequencing

NoV inoculums (GII_A and GII_B) were sequenced at three regions; the polymerase region was sequenced with the primers MJV12 and RegA [38], the ORF1-2 junction with QNIF2d [36] and G2SKR [39] and the capsid region with CapD1, CapD3, and CapC [38]. Sequencing was performed using BigDye v. 3.1 terminator chemistry and analyzed on an ABI PRISM 310 Genetic Analyzer (Applied Biosystems, Foster City, CA, USA). The NoV sequences were assigned using the Norovirus Genotyping Tool [40].

### 4.5. Modelling of Decay Curves

GInaFiT (Geeraerd and Van Impe Inactivation Model Fitting Tool) [41], a freeware add-in for Microsoft Excel 2010, was used for testing different microbial survival models. Models were selected based on the root mean sum of the squared errors (RMSE). The RMSE can be considered as the simplest and most informative measure of goodness-of-fit, both for linear and non-linear models [42]. The model with the lowest RMSE with a comparable experimental precision was considered the best fit. If the same or similar RMSE values were obtained, the less complex model was considered to fit best [41].

In addition to log-linear model (Equation (1)), three non-linear models; log-linear shoulder tail (Equation (2)), Weibull (Equation (3)), and double Weibull (Equation (4)) models were applied to describe the decay patterns. Log-linear model equation is described as:

(1) $$\log_{10}\left(N\right)=\log_{10}\left(N\left(0\right)\right)-\frac{k_{max}t}{\ln\left(10\right)}$$

where *t* is the time, *N* represents the microbial cell density, *N*(0) the initial microbial cell density, and *k_max_* the first order inactivation constant.

The log-linear shoulder tail equation [43] is described as:

(2) $$\log_{10}\left(N\right)=\log_{10}\left[\left(10^{\log_{10}\left(N\left(0\right)\right)}-\;10^{\log_{10}\left(N_{res}\right)}\right)\times e^{-k_{max}t}\;\times \left(\frac{e^{k_{max}S_{1}}}{1+\;\left(e^{k_{max}S_{1}}-1\right)\times e^{-k_{max}t}}\right)+10^{\log_{10}\left(N_{res}\right)}\right]$$

where *N_res_* is the residual population density and *S*_1_ represents shoulder length.

The Weibull model equation [44,45] is described as:

(3) $$\log_{10}\left(N\right)=\log_{10}\left(N\left(0\right)\right)-{\left(\frac{t}{\delta }\right)}^{p}$$

where *δ* is a scale parameter representing the time for achieving 1 log reduction and *p* is a shape parameter.

The double Weibull model equation [46] is described as:

(4) $$\log_{10}\left(N\right)=\log_{10}\left[\frac{10^{\log_{10}\left(N\left(0\right)\right)}}{1+10^{\alpha }}\times \left(10^{-{\left(\frac{t}{\delta_{1}}\right)}^{p}+\alpha }+10^{-{\left(\frac{t}{\delta_{2}}\right)}^{p}}\right)\right]$$

where the subscripts 1 and 2 indicate the two different subpopulations and α is a parameter varying from negative infinity to positive infinity (Equation (5)):

(5) $$\alpha =\log_{10}\left(\frac{f}{1-f}\right)$$

where *f* is the fraction of subpopulation 1 in the population.

### 4.6. Data Analysis

Related-samples Wilcoxon signed rank test was used to assess the statistical significance of differences in log_10_ reductions between temperatures, matrices, and NoV strains. The statistical analyses were conducted using SPSS 24 software for Windows. In statistical calculations, the method detection limit values were used when a below limit of detection (LOD) result was obtained. Differences were considered significant if the *p*-value was <0.05. For the decay rate comparison, T90 and T99.99 (the time to reduce 90% and 99.99% of the initial numbers) were determined using the log-linear decay model. For non-linear decay, the time required to reduce the first log_10_ (TFL) and the time required to reduce fourth log_10_ (T4L) were determined. Only positive samples providing quantitative data were used in the determination of the decay rates.

## Figures and Tables

**Table 1:** Summary of the persistence results using the first-order log-linear and/or non-linear decay models. RMSE = root mean sum of squared error. DW = drinking water. WW = wastewater.

			Log-Linear Model			Non-Linear Model					
T	Water	Virus	*k_max_*	R^2^	RMSE					R^2^	RMSE
3 °C	DW	GII_A	N/A ^1^			N/A					
		GII_B	N/A			N/A					
						*Double Weibull*					
						α	*δ*_1_	*p*	*δ*_2_		
	WW	GII_A	0.06 ± 0.001	0.957	0.30	2.46 ± 0.20	57.06 ± 2.68	2.80 ± 0.44	158.5 ± 21.9	0.996	0.11
		GII_B	0.05 ± 0.002	0.986	0.14	2.63 ± 0.56	50.06 ± 1.94	1.38 ± 0.11	453.3 ± 959.1	0.999	0.05
						*Log-linear shoulder tail*					
						*S*_1_	*k_max_*	Log_10_(*N*_res_)			
		GII_ind	0.02 ± 0.003	0.801	0.20	58.85 ± 3.64	0.23 ± 0.17	2.82 ± 0.05		0.967	0.09
						*Weibull*					
						*δ*	*p*				
21 °C	DW	GII_A	0.01 ± 0.001	0.885	0.22	298.2 ± 8.6	2.63 ± 0.36			0.983	0.09
						*Double Weibull*					
						α	*δ*_1_	*p*	*δ*_2_		
		GII_B	0.02 ± 0.002	0.962	0.28	2.20 ± 0.20	131.6 ± 10.3	3.84 ± 1.24	370.3 ± 25.7	0.995	0.11
						*Log-linear shoulder tail*					
						*S*_1_	*k_max_*	Log_10_(*N*_res_)			
	WW	GII_A	0.04 ± 0.010	0.577	0.80	9.42 ± 0.30	0.54 ± 0.02	4.18 ± 0.01		0.999	0.03
		GII_B	0.06 ± 0.020	0.544	1.35	4.71 ± 0.73	0.59 ± 0.03	2.14 ± 0.04		0.998	0.10
		GII_ind	0.02 ± 0.005	0.636	0.31	8.40 ± 2.25	0.30 ± 0.09	2.48 ± 0.04		0.972	0.10
36 °C	DW	GII_A	0.04 ± 0.003	0.967	0.31	N/A					
		GII_B	0.07 ± 0.004	0.988	0.06	N/A					
	WW	GII_A	0.21 ± 0.010	0.996	0.11	N/A					
		GII_B	0.18 ± 0.010	0.982	0.19	N/A					
		GII_ind	0.05 ± 0.010	0.880	0.07	N/A					

^1^ N/A, not applicable.

**Table 2:** Decay rates of the NoV genome under different temperatures and water matrices. T90 and T99.99 values (days) for log-linear decay, and TFL and T4L (time required to achieve 1 and 4 log_10_ reduction, respectively) values (days) for non-linear decay are presented. DW = drinking water. WW = wastewater.

				Log-Linear		Non-Linear	
T	Water	Virus	Best Fitting Model	T90	T99.99	TFL	T4L
3 °C	DW	GII_A	N/A ^1^	Na^2^	Na	Na	Na
		GII_B	N/A	Na	Na	Na	Na
	WW	GII_A	Double Weibull	38	154	57	185
		GII_B	Double Weibull	45	179	50	569
		GII_ind	Log-linear shoulder	115	461	Na	Na
21 °C	DW	GII_A	Weibull	230	921	298	505
		GII_B	Double Weibull	115	461	132	431
	WW	GII_A	Log-linear shoulder	58	230	14	Na
		GII_B	Log-linear shoulder	38	154	8	21
		GII_ind	Log-linear shoulder	115	461	19	Na
36 °C	DW	GII_A	Log-linear	58	230	N/A	N/A
		GII_B	Log-linear	33	132	N/A	N/A
	WW	GII_A	Log-linear	11	44	N/A	N/A
		GII_B	Log-linear	13	51	N/A	N/A
		GII_ind	Log-linear	46	184	N/A	N/A

^1^ N/A, not applicable; ^2^ Na, not achieved.

**Table 3:** Description of NoV inoculums.

	GII_A	GII_B
Patient age and gender	29 years Female	2 years Female
Assumed exposure	1 April 2011	Not known
Symptoms started	2 April 2011	23–24 February 2012
Stool sample taken	3 April 2011	25 February 2012
Symptoms relieved	4 April 2011	26 February 2012
Storage prior to preparation of inoculum	Immediately at −20 °C (five years and 26 days)	One week outdoors at an average temperature of −4.6 °C (range −20–5.2 °C) [34], including three putative freeze thaw cycles, and then 4 March 2012 at −20 °C (four years, one month and 25 days)

**Table 4:** The physical-chemical properties of the drinking water and wastewater.

	Drinking Water [35]	Wastewater
Turbidity, FTU	0.1	23.15
Color, mg Pt L^−1^	<5	Nd ^1^
pH	7.7	7.03
Conductivity, μS cm^−1^	263	1026
TOC ^2^, mg C L^−1^	2.1	Nd
Free chlorine, mg Cl_2_ L^−1^	0.33	Nd

^1^ Nd, no data; ^2^ TOC, total organic carbon.

## Supplementary Materials

The following are available online at www.mdpi.com/2076-0817/6/4/48/s1; **Table S1.** Literature survey of decay rates of norovirus genome in liquid.
