# Correction: Kauppinen, A.; Miettinen, I.T. Persistence of Norovirus GII Genome in Drinking Water and Wastewater at Different Temperatures. *Pathogens* 2017, *6*(4), 48

**DOI:** 10.3390/pathogens7020045

**Published:** 2018-04-20

**Authors:** Ari Kauppinen, Ilkka T. Miettinen

**Affiliations:** Department of Health Security, Expert Microbiology Unit, National Institute for Health and Welfare, P.O. Box 95, FI-70701 Kuopio, Finland; ilkka.miettinen@thl.fi

The authors wish to make the following corrections to their paper [1]:

Replacing “Log-linear shoulder” in Table 2 with “Log-linear shoulder tail”, and “mmol L^−1^” in Section 4.3 with “µmol L^−1^”.

The authors would like to apologize for any inconvenience caused.

The manuscript will be updated and the original will remain available on the article webpage.
