# Supplementary material for: Persistence of Norovirus GII Genome in Drinking Water and Wastewater at Different Temperatures
Source: Pathogens. 2017 Oct 11;6(4):48. doi: 10.3390/pathogens6040048 (PMC5750572; doi:10.3390/pathogens6040048)
Supplement: Supplementary file 1 [file pathogens-06-00048-s001.pdf]

Supplementary Material

Table S1. Literature survey of decay rates of norovirus genome in liquid.

Matrix	Temperature	Log_{10}/day	T90	T99.99	Reference
Groundwater	4°C	0.01±0.05	100*	400*	[16]
Groundwater	25°C	0.01±0.05	100*	400*	[16]
Surface water	4°C	0.04±0.02	25*	100*	[16]
Surface water	25°C	0.08±0.02	12.5*	50*	[16]
PBS	4°C	0.0086**	116**	465**	[21]
PBS	RT	0.0286**	35**	140**	[21]
PBS	37°C	0.1814**	5.5**	22**	[21]
Wastewater	4°C	0.0087	114.9	460*	[18]
Wastewater	20°C	0.0445	22.5	90*	[18]

*Calculated from log_{10} day^{-1} reduction values reported in the paper; **Calculated from the slope values reported in the paper
